# Supplementary material for: Genetic risk score for insulin resistance based on gene variants associated to amino acid metabolism in young adults
Source: PLoS One. 2024 Feb 29;19(2):e0299543. doi: 10.1371/journal.pone.0299543 (PMC10903913; doi:10.1371/journal.pone.0299543)
Supplement: S1 Appendix — (DOCX) [file pone.0299543.s001.docx]

**Table 1**. **Single nucleotide polymorphisms associated with cardiometabolic risk factors.**

| Gen | Name | Chr | SNP | Allele (reference/variant) | Cardiometabolic risk factors or associated alterations of plasma amino acids | REPORTED risk allele | Reference |
| --- | --- | --- | --- | --- | --- | --- | --- |
| *TAT* | Tyrosine Aminotransferase | 16 | rs74344827 | G/A | Enzyme associated with tyrosine increase. | A | [1] |
| *HGD* | Homogentisate 1,2-Dioxygenase | 3 | rs2255543 | T/A | Gene related to alterations in amino acid and glucose metabolism. | - | [2] |
| *GSTZ1* | Glutathione S-Transferase Zeta 1 | 14 | rs1046428 | T/C | Gene related to alterations in amino acid and glucose metabolism. | - | [2] |
| *GPT* | Glutamic-Pyruvate Transaminase | 8 | rs1063739 | C/A | Gene predictive of T2D, CVD, MetS and IR. | A | [3] |
| *OTC* | Ornithine Transcarbamylase | 38 | rs1800321 | A/G | Hypertension | A | [4] |
| *ASPG* | Asparaginase | 14 | rs1744284 | C/G | - | - | - |
| *HAL* | Histidine Ammonia-Lyase | 12 | rs7297245 | C/T | - | - | - |
| *BCAT2* | Branched Chain Amino Acid Transaminase 2 | 19 | rs11548193 | G/C | Increased BCAA levels in obese subjects. Relationship with IR. | C | [5, 6, 7, 8] |
| *BCKDH* | Branched Chain Keto Acid Dehydrogenase | 19 | rs45500792 | T/G | Increased BCAA levels in obese subjects.  Relationship with MetS and IR. | G | [5, 9] |
| *PRODH* | Proline Dehydrogenase 1 | 22 | rs5747933 | G/T | Associated with high levels of circulating proline. | T | [10, 11] |
| *DLD* | Dihydrolipoamide Dehydrogenase | 7 | rs6943999 | A/T | - | - | - |
| *SHMT1* | Serine Hydroxymethyltransferase 1 | 17 | rs1979277 | G/A | Increases the risk of CVD. | A | [12] |
| *MTR* | 5-Methyltetrahydrofolate-Homocysteine Methyltransferase | 1 | rs1805087 | A/G | Increases the risk of CVD.  Relationship with dyslipidemias. | G | [12, 13, 14] |
| *SLC1A4* | Solute Carrier Family 1 Member 4 | 2 | rs759458 | G/A | Gene related to serum valine levels. | - | [1] |
| *SLC7A9* | Solute Carrier Family 7 Member 9 | 19 | rs1007160 | G/T | - | - | - |
| *PPM1K* | Protein Phosphatase, Mg^2+^/Mn^2+^ Dependent 1K | 4 | rs9637599 | A/C | Association with fasting circulating BCAA levels. Relationship with valine levels. | C | [15, 16] |
| *PPM1K* | Protein Phosphatase, Mg^2+^/Mn^2+^ Dependent 1K | 4 | rs1440581 | T/C | Increased serum BCAA and risk of T2D. | C | [17, 18, 19] |
| *GCKR* | Glucokinase Regulator | 2 | rs1260326 | T/C | Increased serum isoleucine. Association with hyperglycemia and high LDL-C levels. Association with MetS. | T | [19, 20, 21] |

SNP: Single nucleotide polymorphism; CHR: Chromosome; T2D: Type 2 diabetes; CVD: Cardiovascular disease; MetS: Metabolic syndrome; IR: Insulin resistance; BCAA: Branched chain amino acids. LDL-C: Low-density lipoprotein cholesterol.

| Gen | SNP | Homozygotes  n (%) | Heterozygotes  n (%) | Variant Homozygotes  n (%) | HWE  p |
| --- | --- | --- | --- | --- | --- |
| *TAT* | rs74344827 | GG  379 (83.8) | GA  72 (15.9) | AA  1 (0.22) | **0.200^*^** |
| *HGD* | rs2255543 | TT  312 (69) | TA  125 (27.6) | AA  15 (3.31) | **0.570^*^** |
| *GSTZ1* | rs1046428 | TT  168 (37.1) | TC  284 (62.8) | CC  0 (0.0) | < 0.001 |
| *GPT* | rs1063739 | CC  159 (35.1) | CA  218 (48.2) | AA  75 (16.5) | **0.980^*^** |
| *OTC* | rs1800321 | AA  395 (87.3) | AG  32 (7.07) | GG  25 (5.53) | < 0.001 |
| *ASPG* | rs1744284 | CC  203 (44.9) | CG  245 (54.2) | GG  4 (0.88) | < 0.001 |
| *HAL* | rs7297245 | CC  371 (82.1) | CT  76 (16.8) | TT  5 (1.11) | **0.610^*^** |
| *BCAT2* | rs11548193 | GG  381 (84.3) | GC  68 (15) | CC  3 (0.66) | **0.980^*^** |
| *BCKDH* | rs45500792 | TT  402 (88.9) | TG  41 (9.07) | GG  9 (1.99) | < 0.001 |
| *PRODH* | rs5747933 | GG  311 (68.8) | GT  129 (28.5) | TT  12 (2.65) | **0.750^*^** |
| *DLD* | rs6943999 | AA  179 (39.6) | AT  217 (48) | TT  56 (12.4) | **0.430^*^** |
| *SHMT1* | rs1979277 | GG  342 (75.7) | GA  91 (20.1) | AA  19 (4.20) | < 0.001 |
| *MTR* | rs1805087 | AA  303 (67) | AG  122 (27) | GG  27 (6) | < 0.001 |
| *SLC1A4* | rs759458 | GG  327 (72.3) | GA  24 (5.3) | AA  101 (22.3) | < 0.001 |
| *SLC7A9* | rs1007160 | GG  342 (75.7) | GT  97 (21.5) | TT  13 (2.88) | **0.060^*^** |
| *PPM1K* | rs9637599 | AA  240 (53.1) | AC  81 (17.9) | CC  131 (29) | < 0.001 |
| *PPM1K* | rs1440581 | TT  172 (38.1) | TC  213 (47.1) | CC  67 (14.8) | **0.930^*^** |
| *GCKR* | rs1260326 | TT  194 (42.9) | TC  209 (46.2) | CC  49 (10.8) | **0.510^*^** |

**Table 2. Genotypic frequencies of the study subjects (n=452).**

SNP: Single nucleotide polymorphism; HWE: Hardy-Weinberg equilibrium.

**^*^** Hardy-Weinberg equilibrium *p* > 0.05.

**Table 3. Presumed risk alleles of candidate single nucleotide** **polymorphisms used to calculate the GRS based on HOMA-IR values of the study subjects (n=452).**

| Gen | SNP | Homozygotes | Heterozygotes | Variant Homozygotes | Presumed Risk Allele for GRS ^1^ | Reported Risk Allele |
| --- | --- | --- | --- | --- | --- | --- |
|  |  | **GG**  **n=379** | **GA**  **n=72** | **AA**  **n=1** |  |  |
| TAT | rs74344827 | 2.34 (1.74-3.18) | 2.19 (1.66-2.95) | 0.99 (0.99-0.99) | G | A |
|  |  | **TT**  **n=312** | **TA**  **n=125** | **AA**  **n=15** |  |  |
| HGD | rs2255543 | 2.35 (1.75-3.21) | 2.32 (1.69-2.91) | 2.20 (1.67-3.01) | T | - |
|  |  | **CC**  **n=159** | **CA**  **n=218** | **AA**  **n=75** |  |  |
| GPT | rs1063739 | 2.67 (1.88-3.35) | 2.19 (1.61-2.87) | 2.33 (1.80-2.94) | C | A |
|  |  | **CC**  **n=371** | **CT**  **n=76** | **TT**  **n=5** |  |  |
| HAL | rs7297245 | 2.32 (1.71-3.10) | 2.35 (1.83-3.12) | 2.36 (1.52-3.01) | T | - |
|  |  | **GG**  **n=381** | **GC**  **n=68** | **CC**  **n=3** |  |  |
| BCAT2 | rs11548193 | 2.32 (1.72-3.06) | 2.48 (1.79-3.18) | 2.28 (1.78-8.0) | C | C |
|  |  | **GG**  **n=311** | **GT**  **n=129** | **TT**  **n=12** |  |  |
| PRODH | rs5747933 | 2.39 (1.74-3.25) | 2.19 (1.75-2.88) | 2.22 (1.30-2.79) | G | T |
|  |  | **AA**  **n=179** | **AT**  **n=217** | **TT**  **n=56** |  |  |
| DLD | rs6943999 | 2.41 (1.82-3.24) | 2.30 (1.68-2.94) | 2.11 (1.67-2.95) | A | - |
|  |  | **GG**  **n=342** | **GT**  **n=97** | **TT**  **n=13** |  |  |
| SLC7A9 | rs1007160 | 2.39 (1.79-3.19) | 2.19 (1.60-2.76) | 2.02 (1.57-3.73) | G | - |
|  |  | **TT**  **n=172** | **TC**  **n=213** | **CC**  **n=67** |  |  |
| PPM1K | rs1440581 | 2.22 (1.78-2.87) | 2.44 (1.70-3.26) | 2.33 (1.62-3.22) | C | C |
|  |  | **TT**  **n=194** | **TC**  **n=209** | **CC**  **n=49** |  |  |
| GCKR | rs1260326 | 2.31 (1.66-3.05) | 2.36 (1.82-3.22) | 2.26 (1.75-2.97) | T | T |

Data are shown as median (25^th^ - 75^th^ percentile). SNP: Single nucleotide polymorphism; GRS: Genetic Risk Score.

**^1^** The risk allele was selected based on the highest median HOMA-IR (Homeostatic Model Assessment-Insulin Resistance).

**Table 4. Genotype and allelic frequencies stratified by insulin resistance (n=452).**

Data are shown as n (%). SNP: Single nucleotide polymorphism; IR: Insulin resistance.

**^1^**Data are shown as frequencies.

|  |  | Without IR (n=251) | | | | | With IR (n=201) | | | | |
| --- | --- | --- | --- | --- | --- | --- | --- | --- | --- | --- | --- |
|  |  | **Genotype frequencies** | | | **Allelic frequencies^1^** | | **Genotype frequencies** | | | **Allelic frequencies^1^** | |
| Gen | **SNP** | **Homozygotes** | **Heterozygotes** | **Variant**  **Homozygotes** | **Allele** | | **Homozygotes** | **Allele** | **Variant**  **Homozygotes** | **Allele** | |
| TAT | rs74344827 | **GG** | **GA** | **AA** | **G** | **A** | **GG** | **GA** | **AA** | **G** | **A** |
|  |  | 207 (82.5) | 43 (17.1) | 1 (0.40) | 0.91 | 0.09 | 172 (85.6) | 29 (14.4) | 0 | 0.92 | 0.07 |
| HGD | rs2255543 | **TT** | **TA** | **AA** | **T** | **A** | **TT** | **TA** | **AA** | **T** | **A** |
|  |  | 170 (67.7) | 72 (28.7) | 9 (3.60) | 0.82 | 0.18 | 142 (70.6) | 53 (26.4) | 6 (3.0) | 0.84 | 0.16 |
| GPT | rs1063739 | **CC** | **CA** | **AA** | **C** | **A** | **CC** | **CA** | **AA** | **C** | **A** |
|  |  | 73 (29.1) | 139 (55.4) | 39 (15.5) | 0.56 | 0.44 | 86 (42.8) | 79 (39.3) | 36 (17.9) | 0.62 | 0.38 |
| HAL | rs7297245 | **CC** | **CT** | **TT** | **C** | **T** | **CC** | **CT** | **TT** | **C** | **T** |
|  |  | 206 (82.1) | 42 (16.7) | 3 (1.20) | 0.91 | 0.09 | 165 (82.1) | 34 (16.9) | 2 (1.0) | 0.90 | 0.10 |
| BCAT2 | rs11548193 | **GG** | **GC** | **CC** | **G** | **C** | **GG** | **GC** | **CC** | **G** | **C** |
|  |  | 215 (85.7) | 34 (13.5) | 2 (0.80) | 0.92 | 0.08 | 166 (82.6) | 34 (16.9) | 1 (0.50) | 0.91 | 0.09 |
| PRODH | rs5747933 | **GG** | **GT** | **TT** | **G** | **T** | **GG** | **GT** | **TT** | **G** | **T** |
|  |  | 165 (65.7) | 80 (31.9) | 6 (2.40) | 0.82 | 0.18 | 146 (72.6) | 49 (24.4) | 6 (3.0) | 0.85 | 0.15 |
| DLD | rs6943999 | **AA** | **AT** | **TT** | **A** | **T** | **AA** | **AT** | **TT** | **A** | **T** |
|  |  | 93 (37.1) | 124 (49.4) | 34 (13.5) | 0.62 | 0.38 | 86 (42.8) | 93 (46.3) | 22 (10.9) | 0.66 | 0.34 |
| SLC7A9 | rs1007160 | **GG** | **GT** | **TT** | **G** | **T** | **GG** | **GT** | **TT** | **G** | **T** |
|  |  | 184 (73.3) | 58 (23.1) | 9 (3.60) | 0.85 | 0.15 | 158 (78.6) | 39 (19.4) | 4 (2.0) | 0.88 | 0.12 |
| PPM1K | rs1440581 | **TT** | **TC** | **CC** | **T** | **C** | **TT** | **TC** | **CC** | **T** | **C** |
|  |  | 104 (41.4) | 109 (43.4) | 38 (15.1) | 0.63 | 0.37 | 68 (33.8) | 104 (51.7) | 29 (14.4) | 0.60 | 0.40 |
| GCKR | rs1260326 | **TT** | **TC** | **CC** | **T** | **C** | **TT** | **TC** | **CC** | **T** | **C** |
|  |  | 109 (43.4) | 115 (45.8) | 27 (10.8) | 0.66 | 0.34 | 85 (42.3) | 94 (46.8) | 22 (10.9) | 0.66 | 0.34 |

| Model | Gen | SNP | Coefficient (β) | | SD | *p* | R^2^ |
| --- | --- | --- | --- | --- | --- | --- | --- |
|  |  |  | **Unstandardized** | **Standardized** |  |  |  |
| 1  2  3  4  5  6  7 | *TAT*  *HGD*  *GPT*  *HAL*  *BCAT2*  *PRODH*  *DLD*  *SLC7A9*  *PPM1K*  *GCKR*  *TAT*  *HGD*  *GPT*  *HAL*  *BCAT2*  *PRODH*  *DLD*  *SLC7A9*  *PPM1K*  *TAT*  *HGD*  *GPT*  *HAL*  *PRODH*  *DLD*  *SLC7A9*  *PPM1K*  *TAT*  *HGD*  *GPT*  *HAL*  *PRODH*  *DLD*  *SLC7A9*  *TAT*  *HGD*  *GPT*  *PRODH*  *DLD*  *SLC7A9*  *HGD*  *GPT*  *PRODH*  *DLD*  *SLC7A9*  *HGD*  *PRODH*  *DLD*  *SLC7A9* | rs74344827  rs2255543  rs1063739  rs7297245  rs11548193  rs5747933  rs6943999  rs1007160  rs1440581  rs1260326  rs74344827  rs2255543  rs1063739  rs7297245  rs11548193  rs5747933  rs6943999  rs1007160  rs1440581  rs74344827  rs2255543  rs1063739  rs7297245  rs5747933  rs6943999  rs1007160  rs1440581  rs74344827  rs2255543  rs1063739  rs7297245  rs5747933  rs6943999  rs1007160  rs74344827  rs2255543  rs1063739  rs5747933  rs6943999  rs1007160  rs2255543  rs1063739  rs5747933  rs6943999  rs1007160  rs2255543  rs5747933  rs6943999  rs1007160 | 0.099  0.246  0.136  0.087  -0.030  0.358  0.194  0.335  0.027  0.000  0.099  0.246  0.136  0.087  -0.030  0.358  0.194  0.335  0.027  0.100  0.246  0.137  0.085  0.356  0.194  0.335  0.028  0.100  0.246  0.137  0.087  0.357  0.193  0.334  0.101  0.240  0.136  0.352  0.195  0.332  0.240  0.137  0.354  0.194  0.336  0.249  0.345  0.215  0.352 | 0.024  0.087  0.062  0.024  -0.008  0.124  0.085  0.111  0.012  0.000  0.024  0.087  0.062  0.024  -0.008  0.124  0.085  0.111  0.012  0.025  0.087  0.062  0.023  0.123  0.085  0.111  0.013  0.025  0.087  0.063  0.024  0.123  0.085  0.111  0.025  0.085  0.062  0.122  0.086  0.110  0.085  0.063  0.122  0.085  0.112  0.088  0.119  0.094  0.117 | 0.189  0.132  0.104  0.171  0.184  0.137  0.108  0.141  0.103  0.109  0.189  0.132  0.103  0.170  0.184  0.137  0.108  0.141  0.103  0.188  0.132  0.103  0.170  0.136  0.108  0.141  0.103  0.188  0.131  0.103  0.169  0.136  0.107  0.141  0.188  0.131  0.103  0.135  0.107  0.140  0.131  0.103  0.135  0.107  0.140  0.131  0.135  0.106  0.140 | 0.600  0.063  0.190  0.611  0.870  **0.009**  0.075  **0.018**  0.793  0.998  0.599  0.062  0.188  0.610  0.869  **0.009**  0.073  **0.018**  0.792  0.597  0.063  0.187  0.616  **0.009**  0.072  **0.018**  0.786  0.595  0.062  0.184  0.609  **0.009**  0.073  **0.018**  0.592  0.068  0.188  **0.009**  0.070  **0.018**  0.067  0.184  **0.009**  0.071  **0.017**  0.057  **0.011**  **0.044**  **0.012** | 0.027  0.029  0.031  0.034  0.035  0.037  0.035 |

**Table 5. Multiple linear regression models for the choice of genetic risk score for HOMA-IR (n=452).**

SNP: Single nucleotide polymorphism; HOMA-IR: Homeostatic Model Assessment-Insulin Resistance. SD: Standard deviation.

**Table 6. Trend between anthropometric, clinical, and biochemical parameters and the genetic risk score for HOMA-IR (n=452).**

| Characteristic  Weight (kg)  BMI (kg/m^2^)  SBP (mmHg)  DBP (mmHg)  Glucose (mg/dL)  TC (mg/dL)  HDL-C (mg/dL)  LDL-C (mg/dL)  TG (mg/dL)  Insulin (µU/mL)  Leptin (ng/mL)  HOMA-IR | GRS-low  T1 (0.624) | GRS-medium  T2 (0.742) | GRS-high  T3 (0.836) | *p*^1^  0.045^*^  0.046^*^  0.884  0.829  0.021^*^  0.016^*^  0.732  0.081  0.003^*^  <0.001^*^  0.022^*^  <0.001^*^ |
| --- | --- | --- | --- | --- |
|  | n=149 | n=211 | n=92 |  |
|  | 64 (57-74)  23.5 (21.6-26.1)  110 (100-110)  70 (60-80)  78 (73-85)  150 (125-167)  66 (56.7-74.9)  69 (40.1-79)  91 (73-127)  10.8 (8.14-14.2)  13.3 (4.22-17.9)  2.12 (1.52-2.82) | 65 (57-77)  23.7 (21.2-27)  110 (100-110)  70 (60-80)  79 (74-85)  151 (133-173)  67 (58.5-76.2)  61 (44.7-80)  99 (71-133)  12 (9.64-16)  11.6 (4.83-20.7)  2.34 (1.79-3.08) | 68.2 (60.2-76.5)  25.2 (22-27)  110 (100-110)  70 (60-809  80 (75-87.7)  160 (135-179)  64.3 (56-74)  65.8 (47.7-85)  115 (85-156)  13.1 (9.37-17.5)  15.5 (4.80-26.1)  2.51 (1.84-3.50) |  |

Data are shown as median (25^th^ - 75^th^ percentile). GRS: Genetic Risk Score; T1: First tertile; T2: Second tertile; T3: Third tertile; HOMA-IR: Homeostatic Model Assessment - Insulin Resistance; BMI: Body mass index; SBP: systolic blood pressure; DBP: diastolic blood pressure; TC: total cholesterol; HDL-C: High-density lipoprotein cholesterol; LDL-C: Low-density lipoprotein cholesterol; TG: Triglycerides.

**^1^**Differences are based on Jonckheere-Terpstra test.

**^*^** The difference is significant *p* ≤ 0.05.

**Table 7. Trend between amino acids and the genetic risk score for HOMA-IR (n=452).**

| Characteristic  Aspartate (µM)  Glutamate (µM)  Serine (µM)  Histidine (µM)  Glycine (µM)  Threonine (µM)  Arginine (µM)  Alanine (µM)  Tyrosine (µM)  Valine (µM)  Methionine (µM)  Phenylalanine (µM)  Isoleucine (µM)  Leucine (µM)  Lysine (µM)  Proline (µM)  BCAA (µM) | GRS-low  T1 (0.624) | GRS-medium  T2 (0.742) | GRS-high  T3 (0.836) | *p*^1^  0.605  0.688  0.296  0.223  0.064  0.747  0.103  0.131  0.710  0.916  0.100  0.830  0.614  0.867  0.632  0.019^*^  0.929 |
| --- | --- | --- | --- | --- |
|  | n=149 | n=211 | n=92 |  |
|  | 28.5 (22.7-35)  86.9 (72-100)  131 (112-150)  63.2 (39.9-73.6)  272 (213-326)  163 (125-201)  86.3 (74-99)  549 (457-621)  55.1 (47.5-64)  180 (134-229)  70.7 (37.3-94.8)  71 (53.2-86.9)  56.1 (48.4-66)  116 (97-132)  169 (136-202)  191 (128-250)  356 (294-421) | 27 (22.1-33.1)  83.6 (68.4-103)  123 (108-147)  62.5 (41-73.5)  258 (200-326)  155 (119-193)  81.2 (70-93.5)  561 (461-656)  53.8 (46.1-64.1)  180 (135-233)  68 (44.4-97.6)  66.5 (52.3-82.7)  54.9 (45.2-63.6)  116 (94.3-133)  167 (140-199)  175 (123-229)  361 (288-433) | 30 (25.2-35)  89 (73.9-107)  133 (107-150)  58.5 (27-67)  244 (201-293)  164 (120-208)  86.2 (69.5-95.7)  565 (484-669)  56 (46.4-67)  174 (144-228)  85.6 (46-107)  70 (57.3-82.9)  55 (44.5-66.6)  119 (96-134)  169 (149-200)  160 (116-207)  372 (287-426) |  |

Data are shown as median (25^th^ - 75^th^ percentile). GRS: Genetic Risk Score; T1: First tertile; T2: Second tertile; T3: Third tertile; HOMA-IR: Homeostatic Model Assessment - Insulin Resistance; BMI: Body mass index; SBP: systolic blood pressure; DBP: diastolic blood pressure; TC: total cholesterol; HDL-C: High-density lipoprotein cholesterol; LDL-C: Low-density lipoprotein cholesterol; TG: Triglycerides.

**^1^**Differences are based on Jonckheere-Terpstra test.

**^*^** The difference is significant *p* ≤ 0.05.

**Table 8. Serum amino acid concentrations of subjects according to the genetic risk score for HOMA-IR by sex (n=452).**

| Characteristic  Aspartate (µM)  Glutamate (µM)  Serine (µM)  Histidine (µM)  Glycine (µM)  Threonine (µM)  Arginine (µM)  Alanine (µM)  Tyrosine (µM)  Valine (µM)  Methionine (µM)  Phenylalanine (µM)  Isoleucine (µM)  Leucine (µM)  Lysine (µM)  Proline (µM)  BCAA (µM) | GRS-low  T1 (0.624) | | | GRS-medium  T2 (0.742) | | | GRS-high  T3 (0.836) | | *p*  0.005^*^  0.731  0.248  0.730  0.445  0.474  0.014^*^  0.098  0.630  0.947  0.087  0.839  0.021^*^  0.043^*^  0.140  0.427  0.318 |
| --- | --- | --- | --- | --- | --- | --- | --- | --- | --- |
|  | Men  n=83 | Woman  n=66 | *p* | Men  n=115 | Woman  n=96 | *p* | Men  n=43 | Woman  n=49 |  |
|  | 26.8 (22-32.5)  89.4 (72-99.8)  127 (110-145)  63.7 (39.9-72.4)  251 (210-320)  168 (127-197)  81.1 (70.7-99.5)  548 (465-609)  53.9 (47.7-63.6)  186 (146-247)  74.7 (44.9-103)  67.4 (50.4-83.5)  60.3 (51.4-70.3)  124 (105-137)  172 (139-204)  198 (134-263)  373 (308-450) | 31.2 (24-39)  84.4 (71.6-102)  135 (120-168)  62.3 (26.6-74.1)  282 (221-345)  160 (122-213)  92.1 (81.3-100)  552 (441-626)  55.8 (46.8-64.5)  176 (134-212)  44.5 (29.1-80.8)  75.2 (57.8-94.6)  52.2 (43.5-60.6)  111 (89.1-124)  168 (128-191)  189 (120-238)  342 (281-383) | **0.004^*^**  0.659  **0.003^*^**  0.850  0.125  0.963  **0.009^*^**  0.766  0.974  0.116  **0.031^*^**  0.063  **<0.001^*^**  **0.001^*^**  0.119  0.290  **0.010^*^** | 26.2 (21.6-30.8)  83.5 (70.9-106)  122 (108-145)  64.2 (49.6-77.5)  265 (209-330)  160 (121-199)  80.5 (70-93.3)  566 (464-665)  55 (46.4-66.2)  190 (147-247)  79.2 (46.8-99.6)  67 (53-84.9)  58.6 (49.8-65.4)  121 (105-139)  167 (140-194)  185 (129-233)  379 (306-448) | 29.8 (22.8-35.4)  83.8 (65.7-99.5)  124 (107-147)  51.1 (16.8-69.8)  248 (195-310)  152 (115-184)  83.5 (69.8-93.7)  551 (458-650)  53.5 (45.4-63.1)  170 (119-220)  61.7 (37.7-93.4)  66 (52-81)  51.7 (41.5-61.8)  110 (81.6-126)  167 (138-204)  170 (119-214)  342 (241-389) | **0.008^*^**  0.178  0.586  **0.027^*^**  0.169  0.309  0.826  0.591  0.295  **0.022^*^**  0.192  0.862  **0.003^*^**  **0.001^*^**  0.980  0.243  **0.005^*^** | 27.5 (23.2-31.1)  88.2 (78.6-101)  120 (105-147)  58.4 (24.9-72.6)  238 (188-290)  156 (120-194)  80.6 (66.9-99.8)  535 (448-648)  56.5 (46.5-68.3)  174 (134-232)  94.2 (54.7-111)  69.9 (59.1-82.6)  61.6 (47-72.9)  126 (100-137)  175 (152-210)  161 (127-203)  376 (296-426) | 32.7 (27.4-36.7)  90.6 (70.9-112)  137 (108-158)  57.3 (31.1-64.7)  252 (205-298)  173 (119-222)  91.7 (80.7-97.9)  591 (535-676)  55.4 (46.3-64.7)  174 (144-228)  71.6 (43.3-97)  70 (55.8-85.5)  51.4 (43.9-61.5)  107 (96-130)  163 (147-183)  156 (104-217)  346 (286-422) |  |

Data are shown as median (25th - 75th percentile). µM: Micromolar (µmol/L); GRS: Genetic Risk Score; T1: First tertile; T2: Second tertile; T3: Third tertile; HOMA-IR: Homeostatic Model Assessment - Insulin Resistance; BCAA: Branched Chain Amino Acids.

**^1^** Mann-Whitney U test.

***** The difference is significant *p* ≤ 0.05.

**References**

[1] Kettunen J, Tukiainen T, Sarin AP, Ortega-Alonso A, Tikkanen E, Lyytikäinen LP, et al. Genome-wide association study identifies multiple loci influencing human serum metabolite levels. Nat Genet 2012;44:269–76. https://doi.org/10.1038/ng.1073.

[2] Wang J, Chang H, Su M, Qiao Y, Sun H, Zhao Y, et al. Identification of HGD and GSTZ1 as Biomarkers Involved Metabolic Reprogramming in Kidney Renal Clear Cell Carcinoma. Int J Mol Sci 2022;23. https://doi.org/10.3390/IJMS23094583.

[3] Sookoian S, Pirola CJ. Liver enzymes, metabolomics and genome-wide association studies: from systems biology to the personalized medicine. World J Gastroenterol 2015;21:711–25. https://doi.org/10.3748/WJG.V21.I3.711.

[4] Dumont J, Meroufel D, Bauters C, Hansmannel F, Bensemain F, Cottel D, et al. Association of ornithine transcarbamylase gene polymorphisms with hypertension and coronary artery vasomotion. Am J Hypertens 2009;22:993–1000. https://doi.org/10.1038/AJH.2009.110.

[5] Guizar-Heredia R, Tovar AR, Granados-Portillo O, Pichardo-Ontiveros E, Flores-López A, González-Salazar LE, et al. Serum amino acid concentrations are modified by age, insulin resistance, and BCAT2 rs11548193 and BCKDH rs45500792 polymorphisms in subjects with obesity. Clin Nutr 2021;40:4209–15. https://doi.org/10.1016/J.CLNU.2021.01.037.

[6] Vargas-Morales JM, Guizar-Heredia R, Méndez-García AL, Palacios-Gonzalez B, Schcolnik-Cabrera A, Granados O, et al. Association of BCAT2 and BCKDH polymorphisms with clinical, anthropometric and biochemical parameters in young adults. Nutr Metab Cardiovasc Dis 2021;31:3210–8. https://doi.org/10.1016/J.NUMECD.2021.07.011.

[7] González-Salazar LE, Granados-Portillo O, Medina-Vera I, Pichardo-Ontiveros E, Vigil-Martínez A, Guizar-Heredia R, et al. Effect of the *BCAT2* polymorphism (rs11548193) on plasma branched-chain amino acid concentrations after dietary intervention in subjects with obesity and insulin resistance. Br J Nutr 2021:1–12. https://doi.org/10.1017/S0007114521002920.

[8] Teslovich TM, Kim DS, Yin X, Stančáková A, Jackson AU, Wielscher M, et al. Identification of seven novel loci associated with amino acid levels using single-variant and gene-based tests in 8545 Finnish men from the METSIM study. Hum Mol Genet 2018;27:1664–74. https://doi.org/10.1093/hmg/ddy067.

[9] Serralde-Zúñiga AE, Guevara-Cruz M, Tovar AR, Herrera-Hernández MF, Noriega LG, Granados O, et al. Omental adipose tissue gene expression, gene variants, branched-chain amino acids, and their relationship with metabolic syndrome and insulin resistance in humans. Genes Nutr 2014;9:1–10. https://doi.org/10.1007/s12263-014-0431-5.

[10] Imaizumi A, Adachi Y, Kawaguchi T, Higasa K, Tabara Y, Sonomura K, et al. Genetic basis for plasma amino acid concentrations based on absolute quantification: a genome-wide association study in the Japanese population. Eur J Hum Genet 2019;27:621–30. https://doi.org/10.1038/S41431-018-0296-Y.

[11] Koshiba S, Motoike IN, Saigusa D, Inoue J, Aoki Y, Tadaka S, et al. Identification of critical genetic variants associated with metabolic phenotypes of the Japanese population. Commun Biol 2020;3. https://doi.org/10.1038/s42003-020-01383-5.

[12] Poddar R. Hyperhomocysteinemia is an emerging comorbidity in ischemic stroke. Exp Neurol 2020;336:113541–113541. https://doi.org/10.1016/J.EXPNEUROL.2020.113541.

[13] Li WX, Lv WW, Dai SX, Pan ML, Huang JF. Joint associations of folate, homocysteine and MTHFR, MTR and MTRR gene polymorphisms with dyslipidemia in a Chinese hypertensive population: a cross-sectional study. Lipids Health Dis 2015;14:101–101. https://doi.org/10.1186/S12944-015-0099-X.

[14] Lei H, Song XM, Zhu WL, Li Y. Plasma homocysteine and gene polymorphisms associated with the risk of hyperlipidemia in northern Chinese subjects. Biomed Environ Sci 2008;21:514–20. https://doi.org/10.1016/S0895-3988(09)60011-8.

[15] Mahendran Y, Jonsson A, Have CT, Allin KH, Witte DR, Jørgensen ME, et al. Genetic evidence of a causal effect of insulin resistance on branched-chain amino acid levels. Diabetologia 2017;60:873–8. https://doi.org/10.1007/S00125-017-4222-6/TABLES/1.

[16] Kettunen J, Demirkan A, Würtz P, Draisma HHM, Haller T, Rawal R, et al. Genome-wide study for circulating metabolites identifies 62 loci and reveals novel systemic effects of LPA. Nat Commun 2016;7:11122–11122. https://doi.org/10.1038/NCOMMS11122.

[17] Hu W, Liu Z, Yu W, Wen S, Wang X, Qi X, et al. Effects of *PPM1K* rs1440581 and rs7678928 on serum branched-chain amino acid levels and risk of cardiovascular disease. Ann Med 2021;53:1316–26. https://doi.org/10.1080/07853890.2021.1965204.

[18] Xuan L, Hou Y, Wang T, Li M, Zhao Z, Lu J, et al. Association of branched chain amino acids related variant rs1440581 with risk of incident diabetes and longitudinal changes in insulin resistance in Chinese. Acta Diabetol 2018;55:901–8. https://doi.org/10.1007/S00592-018-1165-4.

[19] Lotta LA, Scott RA, Sharp SJ, Burgess S, Luan J, Tillin T, et al. Genetic Predisposition to an Impaired Metabolism of the Branched-Chain Amino Acids and Risk of Type 2 Diabetes: A Mendelian Randomisation Analysis. PLoS Med 2016;13. https://doi.org/10.1371/journal.pmed.1002179.

[20] Burgess S, Malik R, Liu B, Mason AM, Georgakis MK, Dichgans M, et al. Dose-response relationship between genetically proxied average blood glucose levels and incident coronary heart disease in individuals without diabetes mellitus. Diabetologia 2021;64:845–9. https://doi.org/10.1007/S00125-020-05377-0.

[21] Zahedi AS, Akbarzadeh M, Sedaghati-Khayat B, Seyedhamzehzadeh A, Daneshpour MS. GCKR common functional polymorphisms are associated with metabolic syndrome and its components: a 10-year retrospective cohort study in Iranian adults. Diabetol Metab Syndr 2021;13:20–20. https://doi.org/10.1186/S13098-021-00637-4.
